# Mental Health Disturbance after a Major Earthquake in Northern Peru: A Preliminary, Cross-Sectional Study

**DOI:** 10.3390/ijerph19148357

**Published:** 2022-07-08

**Authors:** Mario J. Valladares-Garrido, Luis E. Zapata-Castro, Helena Domínguez-Troncos, Abigaíl García-Vicente, Darwin A. León-Figueroa, J. Pierre Zila-Velasque, Pamela Grados-Espinoza, David Astudillo-Rueda, C. Ichiro Peralta, Cristian Díaz-Vélez

**Affiliations:** 1 Vicerrectorado de Investigación, Universidad Norbert Wiener, Lima 15046, Peru; 2 Instituto de Evaluación de Tecnologías en Salud e Investigación-IETSI, EsSalud, Lima 15072, Peru; cdiazv3@upao.edu.pe; 3 Hospital Regional Lambayeque, Chiclayo 14012, Peru; 4 School of Medicine, Universidad Nacional de Piura, Piura 20002, Peru; 0902017015@alumnos.unp.edu.pe (L.E.Z.-C.); 0902018007@alumnos.unp.edu.pe (H.D.-T.); 0902019007@alumnos.unp.edu.pe (A.G.-V.); 5 Sociedad Científica de Estudiantes de Medicina de la Universidad Nacional de Piura, Piura 20002, Peru; 6 Emerging Diseases and Climate Change Research Unit, Universidad Peruana Cayetano Heredia, Lima 15013, Peru; darwin_leon@usmp.pe; 7 School of Medicine, Universidad de San Martín de Porres, Chiclayo 14012, Peru; 8 Facultad de Medicina Humana, Universidad Nacional Daniel Alcides Carrión, Pasco 19001, Peru; jzilav@undac.edu.pe (J.P.Z.-V.); pgradose@undac.edu.pe (P.G.-E.); 9 Red Latinoamericana de Medicina en la Altitud e Investigación (REDLAMAI), Pasco 19001, Peru; 10 School of Medicine, Universidad César Vallejo, Piura 20001, Peru; aastudilloru28@ucvvirtual.edu.pe; 11 School of Medicine, Universidad Nacional Federico Villarreal, Lima 15088, Peru; 2015027078@unfv.edu.pe; 12 School of Medicine, Universidad Privada Antenor Orrego, Trujillo 13008, Peru

**Keywords:** depression, anxiety, earthquakes, Peru

## Abstract

Little has been studied in Peru on the mental health repercussions after a major earthquake. We aimed to explore the factors associated with depressive and anxiety symptoms in people who experienced a 6.1 magnitude earthquake in Piura, Peru, on 30 July 2021. A preliminary cross-sectional study was conducted in the general population between August–September 2021. An online questionnaire was provided using PHQ-9, GAD-7, and other relevant measures. Generalized linear models were applied. Of the 177 participants, the median age was 22 years, the majority were female (56%), and many experienced depressive (52%) or anxiety symptoms (52%). Presence of depressive symptoms was associated with a personal history of mental disorder, moderate housing damage, social/material support from politicians, moderate food insecurity, and insomnia. Presence of anxiety symptoms was associated with physical injury caused by the earthquake, mild food insecurity, and insomnia. The development of depressive and anxiety symptoms following the 2021 earthquake experienced in Piura depended on multiple individual and socioeconomic factors. Additional studies should reinforce the factors identified here given the methodological limitations, such as the study design, sampling method, and sample size. This would lead to effective intervention measures to mitigate the impact of earthquakes on mental health.

## 1. Introduction

Depression and anxiety are the most prevalent psychiatric conditions worldwide in the general population. Both conditions can be triggered by stressors at the individual (e.g., distress, insomnia), social (e.g., discrimination, isolation), and environmental level (e.g., climate change, natural disasters) [1]. The experience of natural disasters may have negative effects on mental health, favoring the development of psychiatric disorders in the short- and long-term [2]. Worldwide, it has been reported that between 10 and 40% of survivors of seismic disasters developed depression [3,4,5,6,7,8,9], while 20–50% were at increased risk of developing anxiety [3,9,10].

Previous studies have identified individual factors associated with depression and anxiety in diverse populations after the occurrence of an earthquake. For depression, controversial results have been observed for age. A meta-analysis of 28 studies in the general Haitian population surviving the 2010 earthquake found that younger people had a higher risk of depression [11], but studies in Mexico after the 2017 earthquake and Nepal after the 2015 earthquakes observed that depression was more common at older ages [8,12]. Being a student (compared to other activities) may reduce the level of depressive symptoms, as demonstrated in a study in 290 New Zealand students after the 2010 earthquake [13]. However, studies in 1335 Haitian adults after the 2010 earthquake [4] and in 316 Ecuadorian adolescents after the 2016 earthquake [9] showed opposite results. Furthermore, previous episodes of mental disorders, such as post-traumatic stress disorder (PTSD) and insomnia, may increase the prevalence of depression. This is evidenced by Chinese studies conducted on adult and adolescent survivors of the 2017 Sichuan [14], 2013 Lushan [15], and 2008 Wenchuan [16,17] earthquakes. Moreover, having a family member injured in an earthquake can increase the risk of depression, but the evidence is not consistent. A study conducted in 62 Nepalese children and adolescents after the 2015 earthquakes [18] found an increased risk of depression in those who had injured family members. Conversely, the aforementioned study in Ecuador [9] showed no significant differences. Nonetheless, facing the seismic event with a resilient attitude seems to prevent the development of depressive symptoms. This has been demonstrated by a Japanese study on 241 survivors of the 2011 Fukushima earthquake [19] and the previously mentioned study on the Lushan population [15].

There are situations related to the seismic event that also favors the development of depression. It has been shown that housing damage after an earthquake may increase the rates of depressive symptoms in the Nepalese general population [12], but not in the Ecuadorian adolescents [9]. Social and/or material support (defined as help provided by others in the form of emotional, financial, or food supply) after an earthquake may also generate particular results, since a study in 312 Korean older adults showed lower rates of depression after the 2017 earthquake; but, a study of 1783 older Chinese survivors of the 2008 Sichuan earthquake found higher rates of depressive symptoms [20].

For anxiety, there are similar factors than that reported in studies on depression. For example, individual physical injury caused by an earthquake may trigger anxiety symptoms, as shown in 6132 Chinese adolescent students affected by the 2013 Lushan earthquake [21], but not in 277 Indonesian adult survivors of the 2009 Padang earthquake [22]. Insomnia may also be linked to anxiety after an earthquake, as observed in similar natural disasters, such as the Haiyan typhoon in 361 Filipino survivors [23].

Depression and anxiety are commonly reported after an earthquake. However, previous findings are from studies focused on children and adolescents [6,10], which have not included all the affected cities [4,5], and have assessed only chronic post-traumatic stress disorder (PTSD) several months or years after the catastrophe [4,7]. In addition, studies in Peru have focused on assessing only PTSD [24,25]. Therefore, it is important to understand how depression and anxiety develop in particular settings and what enhances their onset, understanding that the antecedents are still very specific to some populations and the stressors to which they are exposed are unique to the regions they inhabit. This is the case of Piura, a middle-income Peruvian region that is known to experience climatological disasters caused by the Niño Costero, earthquakes due to the “Ring of Fire”, and, more recently, the mental health detriment related to the COVID-19 pandemic.

This preliminary study aimed to explore the factors associated with the presence of anxiety and depressive symptoms in people early affected by the 6.1 magnitude earthquake in Piura, Peru, that occurred on 30 July 2021.

## 2. Materials and Methods

### 2.1. Study Design and Population

A preliminary cross-sectional study was conducted using a virtual survey during August–September 2021, after the earthquake of 6.1 magnitude on the Richter scale that occurred on 30 July 2021. The population consisted of the residents of Piura, which, according to the National Institute of Statistics and Informatics (INEI), had a population of 2,047,954 inhabitants in 2021. Inclusion criteria were people who self-reported having experienced the earthquake and resided in one of the 38 districts of Piura declared in a state of emergency due to the impact of the seismic event.

A sample size was calculated considering a 12% expected prevalence, a confidence level of 95%, a margin of error of 5%, and a rejection rate of 10%. Therefore, a sample size of 179 participants was obtained. Finally, the final sample consisted of 177 participants. A non-probabilistic snowball sampling method was applied.

### 2.2. Procedure

The virtual survey was disseminated through different social networks, which were aimed at the enrollment of the population of Piura in general. Likewise, visibility was sought in different health institutions, universities, local media, and social networks (Facebook, Twitter, WhatsApp, Instagram, etc.). Through this dissemination, the link to the survey was shared; subsequently, data were recorded in the data entry system REDCap, to facilitate the validation and quality control of data entry.

### 2.3. Outcomes

**Anxiety symptoms (GAD-7):** This questionnaire has 7 items with a Likert scale 0–3, which evaluate anxiety symptoms in the last two weeks [26]. It has been validated in Spanish-speaking populations, with a Cronbach’s alpha of 0.94 [27]. Using a cut-off point greater than 10 points, it has a sensitivity of 97%, a specificity of 100%, a positive predictive value >99%, and a negative predictive value of 0.833 [28]. It classifies the absence of anxiety with a score of 0–4 points, mild anxiety with 5–9 points, moderate anxiety with 10–14 points, and severe anxiety with 15–21 points [29]. The alpha coefficient for this study was 0.93.

**Depressive symptoms (PHQ-9):** This questionnaire is composed of 9 items with a Likert scale of 0–3 points [30]. It has been validated in primary care for Latino populations, presenting excellent psychometric properties, with a Cronbach’s alpha of greater than 0.80, a sensitivity of 83%, and a specificity of 82%, taking as a cut-off point a score greater than or equal to seven [31,32]. It has also been validated in the Peruvian population (*n* = 30,449) through an analysis of secondary data from the 2016 ENDES survey, finding a useful invariance for comparison of groups [31]. It is classified as minimal depression level with 0–4 points, mild with 5–9 points, moderate with 10–14 points, moderate-severe with 15–19 points, and severe with 20–27 points [30]. The alpha coefficient for this study was 0.92.

### 2.4. Exposures

**Resilience (CD-RISC):** This questionnaire has 10 items with a Likert scale of 0–4 points [33]. It has been validated in Spanish-speaking healthcare workers, workers in different occupational fields, and young Spanish adults [34,35,36]. It has excellent psychometric properties, with a Cronbach’s alpha of greater than 0.80, a sensitivity of 70%, and a specificity of 68.2% in the discrimination of health personnel with depression, using a score less than or equal to 23 as the cut-off point [33,34,35,36]. The alpha coefficient for this study was 0.95.

**Insomnia (ISI):** This questionnaire consists of 7 items with a Likert scale of 0–4 points [37]. Its use has been validated in the general Spanish-speaking population, with an adequate internal consistency, with a Cronbach alpha of 0.82 [38]. It has also been used to study insomnia in Latino communities residing in the United States (2156 participants) [39]. The instrument classifies the absence of insomnia with a score of 0–7, insomnia below the threshold with 8–14 points, moderate insomnia with 15–21 points, and severe insomnia with 22–28 points [40]. The alpha coefficient was 0.89 for this study.

**Food Security Questionnaire (HFIAS):** This questionnaire consists of nine items with a Likert scale from 1 to 3. It has three domains, covering anxiety and uncertainty about food supply in the household, food quality and insufficient food intake, and physical consequences [41]. Following the FANTA-III (Food and Nutrition Technical Assistance) rubric, mild AI presents with scores of 2–3 on item one, 1–3 on item two, or one on items three or four [41]. Moderate AI presents with scores of 2–3 on items three or four, or 1–2 on items five or six, and severe AI with scores of three on items five or six, or 1–3 on items seven, eight and nine [41]. This scale has been validated for Spanish-speaking Latino populations [41]. The Cronbach’s alpha coefficient was 0.94 for this study.

In the general information questionnaire, data were obtained on age (continuous), sex, marital status, educational level, current job, household income in soles, religion, family members in the household, substance use (alcohol, tobacco), comorbidity, personal and family history of mental health, place where they were when the earthquake occurred, events generated by the earthquake (nervous breakdown, physical injury, family member with physical injury), housing damage, job loss, and social/material support. Nervous breakdown was defined as a sudden feeling of distress that has appeared after the seismic event.

### 2.5. Statistical Analysis

Survey data were organized in Microsoft Excel and analyzed in Stata v.17 (College Station, TX, USA: StataCorp LL).

In the descriptive analysis, the mean/median and standard deviation/25–75th percentile were used to describe numerical variables, depending on the data distribution. For categorical variables, absolute and relative frequencies were reported.

In the bivariate analysis, the association between depressive and anxiety symptoms and study covariates was evaluated. The chi-square test of independence was used for categorical variables and the Student’s *t* test/Mann–Whitney U test for numerical variables, depending on the assumptions of normality and homoscedasticity.

In the simple and multiple regression analysis, generalized linear models were designed. Variables that were statistically significant in the simple regression model were selected for the multiple regression analysis. The Poisson distribution family, log link function, and robust variance were used to estimate prevalence ratios (PR) with 95% confidence intervals. *p* values < 0.05 were considered statistically significant.

### 2.6. Ethical Considerations

The research protocol was approved by the Institutional Research Ethics Committee of the Norbert Wiener University, No. 1495–2021. Informed consent was obtained from each participant, and the data were anonymous, coded, and confidential.

## 3. Results

Table 1 shows the characteristics of the 177 study participants. The median age was 22 years, with an age of 20 at the 25th percentile and 29 at the 75th percentile. The majority were female (*n* = 98, 56%), single (*n* = 140, 79%), and with a higher level of university education (*n* = 113, 64%). Almost half were students (*n* = 98, 55%) and received an income of less than 2000 soles (Peruvian currency). Most did not report drinking alcohol, smoking, having any comorbidity, or any personal or family history of mental problems. Almost the majority were at home when they experienced the earthquake (*n* = 134, 76%) and did not suffer physical (*n* = 172, 97%) or material damage (*n* = 140, 79%). One third showed some degree of food insecurity (*n* = 55, 31%). Less than half experienced some degree of insomnia (*n* = 83, 44%) and high resilience (*n* = 71, 41%). Half of the students experienced some degree of anxiety (*n* = 92, 52%) and depressive symptoms (*n* = 92, 52%).

The presence of depressive symptoms is described according to the general characteristics of the sample in Table 2. The median age was lower in participants who experienced depressive symptoms (21 years depressive vs. 23 years non-depressive, *p* = 0.006). More than half of the single individuals experienced depressive symptoms (*n* = 78, 56%, *p* = 0.023). Most individuals with a personal history of mental problems experienced depressive symptoms (*n* = 11, 92%, *p* = 0.004). Individuals who suffered some degree of damage to their housing more frequently experienced depressive symptoms (46% no damage vs. 71% moderate damage, *p* = 0.024). The presence of depressive symptoms was more frequent in people who experienced some degree of food insecurity (81% severe insecurity vs. 45% no insecurity, *p* < 0.001). A low level of low resilience presented a higher frequency of presence of depressive symptoms (65% low resilience vs. 31% high resilience, *p* < 0.001).

The presence of anxiety symptoms is similarly described in Table 2. The median age was lower in participants who experienced anxiety symptoms (21 years anxiety vs. 22 years no anxiety, *p* = 0.042). Home workers had a higher frequency of presence of anxiety symptoms (89% home worker vs. 54% student, *p* = 0.043). All those who suffered any physical injury experienced anxiety symptoms (*n* = 5, 100%, *p* = 0.029). The presence of some degree of food insecurity was associated with a higher frequency of presence of anxiety symptoms (*n* = 81% severe insecurity vs. 44% no insecurity, *p* = 0.012). The presence of some degree of insomnia was associated with a higher frequency of presence of anxiety symptoms (100% severe insomnia vs. 31% no insomnia, *p* < 0.001). The manifestation of a low level of resilience was associated with a higher frequency of presence of anxiety symptoms (64% low resilience vs. 34% high resilience, *p* < 0.001).

Regression models were developed to explore the presence of factors associated with depressive and anxiety symptoms after the earthquake. Regarding depressive symptoms, being a student (adjusted prevalence ratio aPR = 0.60, 95% CI = 0.44–0.82), or having another type of job (aPR = 0.50, 95% CI = 0.28–0.92) were independently associated with a lower frequency of depressive symptoms. Having a personal history of mental problems was independently associated with a higher frequency of presence of depressive symptoms (aPR = 1.88, 95% CI = 1.33–2.67). Having a family member with physical injury was independently associated with a lower frequency of presence of depressive symptoms (aPR = 0.54, 95% CI = 0.40–0.74). Having suffered moderate housing damage was independently associated with a higher frequency of presence of depressive symptoms (aPR = 2.02, 95% CI = 1.12–3.65). Social/material support received by politicians was independently associated with a higher frequency of presence of depressive symptoms (aPR = 2.33, 95% CI = 1.22–4.45). A moderate level of food insecurity was independently associated with a higher frequency of presence of depressive symptoms (aPR = 1.73, 95% CI = 1.07–2.80). The presence of some degree of insomnia was independently associated with a higher frequency of depressive symptoms (severe insomnia: aPR = 2.74, 95% CI = 1.83–4.11). A high level of resilience was independently associated with a lower frequency of depressive symptoms (aPR = 0.53, 95% CI = 0.42–0.68). More information in Table 3.

Regarding anxiety symptoms, having suffered a physical injury was independently associated with a higher frequency of presence of anxiety symptoms (aPR = 2.69, 95% CI = 1.85–3.93). Mild food insecurity was independently associated with a higher frequency of presence of anxiety symptoms (aPR = 1.49, 95% CI = 1.09–2.03). The presence of some degree of insomnia was independently associated with a higher frequency of presence of anxiety symptoms (severe insomnia: aPR = 3.29, 95% CI = 2.55–4.24). A high level of resilience was independently associated with a lower frequency of presence of anxiety symptoms (aPR = 0.62, 95% CI = 0.52–0.75). More information in Table 4.

## 4. Discussion

### 4.1. Factors Associated with Depressive Symptoms

We found that the older the age, the lower the prevalence of depressive symptoms. This is consistent with a study in survivors of an earthquake in Haiti [4]. It is also similar than that of a meta-analysis, in which adults had a lower prevalence of depressive symptoms compared to children and adolescents [11]. However, this contrasts with what has been described in the adult population of Mexico, who, after 45 years of age, presented a higher prevalence of depression [8]. This also differs with what was found in Nepal after the 2015 earthquake, in which, as age increased, the mean depression score also increased (*p* = 0.017) [12]. This association could be due to the fact that adults may have received psychological support or symptoms were reduced by the sense of control that adults have, which is a protective factor [11]. In our study, however, most of the participants were young (mean age = 22 years) since the surveys were disseminated through the Internet and social networks, which are frequently accessed by this group.

Students presented a lower prevalence of depressive symptoms compared with people who reported being a worker. This is similar to that reported in graduate students, who presented a lower prevalence of depression after seismic events [13]. However, it differs from a study of young students in Haiti, as they presented a higher prevalence of depressive symptoms after the 2010 earthquake [4]. As was the case in Ecuador, in which high school students showed high levels of depression after the 2016 earthquake [9]. The finding of the present study could be explained by the likely intervention that universities or higher education institutions were able to provide to students. These environments provide good community resources that can monitor mental symptoms and offer safe psychosocial and supportive interventions after a disaster [42]. In addition, students can get support from family and society, both of which are resources for better mental health [15].

This study found that those whose family members had some physical injury due to the earthquake experienced fewer depressive symptoms. This is consistent with a study in Ecuador, in which no statistically significant differences were found between depression and physical injury of family members due to the earthquake [9]. Conversely, it differs from what was found in Nepalese children and adolescents who had a family member or friend seriously injured or killed as a result of the earthquake, who presented significantly higher scores for depressive symptoms [18]. This association could be due to the fact that a person witnessing a family member being seriously injured may experience intense fear, and this may or may not be a predictor of depression [43].

Moderate housing damage due to the earthquake doubled the prevalence of depressive symptoms. This is similar to another study conducted in Nepal, which showed a significant association between loss of resources (such as housing) due to earthquake damage and more severe depressive symptoms [12]. This is in contrast to a study that found no statistically significant differences between depression and housing damage from the 2016 Ecuador earthquake [9]. This association could be due to the feeling of hopelessness that people have when they observe the damage caused to their homes [14] and are often no longer habitable.

Additionally, it was found that moderate food insecurity is associated with a 73% increase in the prevalence of depressive symptoms. This is consistent with a study that found that 2 out of 5 adults with low food security have a 41.7% increased prevalence of depression and among adults with very low food security there is a 48.1% increased prevalence of depression [44]. This is also similar with a study that found that very low food security was associated with a 7.49-fold higher prevalence of depression (95% CI: 5.52–10.80) [45]. The association of this study indicates that food insecurity may influence nutrition and physical and mental health. This would generate uncertainty about the ability to maintain and acquire sufficient food, thus provoking a stress response that contributes to depression [46].

Having any type of insomnia was associated with a higher prevalence of depressive symptoms, mainly severe clinical insomnia. This is consistent with a study in China earthquake survivors with sleep disturbances such as insomnia; it was observed that use of sleep medications were significantly associated with depression (p < 0.001) [16]. It is also similar to what was found in Chinese adolescents after the earthquake, in which sleep disturbance was significantly associated with an increased risk of depressive symptoms (OR = 1.51; 95% CI, 1.14–2.02) [17].

### 4.2. Factors Associated with Anxiety

We found that participants who reported a physical injury caused by the earthquake experienced a higher prevalence of anxiety. This is consistent with a study that found an anxiety prevalence of 127% in participants who presented physical injury due to a major earthquake [21]. However, it differs with a study that found a 106% prevalence of anxiety in earthquake-injured subjects compared to uninjured subjects during a 6-month follow-up period [22]. The association found in this study suggests that an earthquake may have a long-term impact on mental health [47].

Mild food insecurity increases the prevalence of anxiety by 49%. This is consistent with a study that found that 2 out of 5 adults with low and very low food security have higher rates of anxiety prevalence [44]. Another study reported that 39% of participants scored positive for anxiety and 44% presented food insecurity (17% low food security and 27% very low food security). Furthermore, in comparison to food-secure individuals, those with very low food security had a 7.49-fold higher prevalence of anxiety [45]. The association found in the present study could be due to a consequence of limited resources that affects many households around the world, causing malnutrition. It not only influences nutrition and physical health, but can also affect mental health by generating anxiety due to the lack of affordable and culturally appropriate food, along with the inability to feed themselves and their families [46].

Presenting subclinical, moderate, and severe clinical insomnia was associated with a higher prevalence of anxiety symptoms. This is consistent with the literature, since multiple studies have confirmed the presence of sleep disturbances, such as insomnia, in adult survivors of natural disasters [23]. In addition, it has been reported that those at high risk of insomnia are 9.8 times more likely to have anxiety, compared to subjects without insomnia [48,49]. The two conditions appear to influence each other over time, and there is increasing evidence that their relationship is probably one of reverse causation [50], because anxiety, caused by the internalization of emotions, predisposes to insomnia and could also intensify depressive symptoms [51]. Given that there is a high comorbidity between these previously described conditions [51], poor sleep quality tend to increase the levels of anxiety [52]. Finally, insomnia cannot be attributed to a single etiology, so a multifactorial and individualized approach is necessary for each affected patient [51]. The results of this study suggest that insomnia may have different implications and consequences, including different patterns of anxiety [48].

### 4.3. Implications of Findings in Mental Health

These preliminary results will be useful for the implementation and improvement of mental health programs in the event of future earthquakes. Community centers should be strengthened with financial support, implementation of protocols for emergency response, and individual assessment for timely and rapid detection of mental health disorders. Furthermore, this population is exposed to particular, unforeseen events, such as the El Niño phenomenon [53], the geographic location in the “Ring of Fire” [54,55], and the recent COVID-19 pandemic. Therefore, contingency plans need to be adapted to this specific context.

### 4.4. Limitations and Strengths

First, the number of participants was small and not as expected based on the sample size calculation. We could not increase this number since the PHQ-9 and GAD-7 results may have been altered if measured over a longer period after the event. In this sense, we also tried to avoid recall bias given that some participants might have forgotten the experience due to mental health care. Therefore, our findings are considered preliminary and further studies should improve the statistical power and sample design. In addition, the cross-sectional design does not allow us to identify causal relationships between the study variables. Measurement bias may also occur since the questionnaires were self-administered. There are other important variables that may have influenced on the outcomes, such as vulnerability of the individual, social networks, and quality of housing. Another limitation is that the results cannot be extrapolated to other Peruvian regions because the event occurred in a particular geographical location, with a particular seismic magnitude. However, most importantly, the non-probabilistic sampling method limits the inference of the data. Therefore, we could not provide valid prevalence estimates and caution is needed when interpreting the results. Despite these limitations, this preliminary study provides novel information with the use of validated instruments to obtain a variety of characteristics that may trigger mental problems. Our findings may serve to lay the groundwork for the development of further, better-designed research, which will ultimately drive the design of effective preventive strategies.

## 5. Conclusions

Two months after the 2021 earthquake occurred in Piura, depressive symptoms were more likely to occur in people with a history of mental disorder, family members injured by the earthquake, moderate housing damage, social/material support from politicians, moderate food insecurity, or with any severity of insomnia symptoms. Older age, being a student, and high resilience reduced the prevalence of depressive symptoms. In addition, anxiety symptoms were more likely to develop in individuals with physical injury caused by the earthquake, mild food insecurity, or with any severity of insomnia symptoms. As with depressive symptoms, high resilience reduced the prevalence of anxiety symptoms. Our findings contribute to better understand these factors in the context of an earthquake. However, they must be interpreted cautiously given the methodological limitations, such as the cross-sectional design, non-probabilistic sampling method, and small sample size. More evidence overcoming these limitations is needed in order to improve local interventions and prevent mental health consequences.

## Figures and Tables

**Table 1:** Characteristics of the participants (*n* = 177).

Characteristics		N (%)
Age (years) *		22 (20–29)
**Sex**		
	Female	98 (56.0)
	Male	77 (44.0)
**Marital status**		
	Single	140 (79.1)
	Married	25 (14.1)
	Cohabitant	10 (5.7)
	Divorced	1 (0.6)
	Separated	1 (0.6)
**Level of education**		
	Secondary	46 (26.0)
	Non-university higher education	18 (10.2)
	University higher education	113 (63.8)
**Current job**		
	Worker	50 (28.3)
	Household worker	9 (5.1)
	Student	98 (55.4)
	Unemployed	7 (4.0)
	Others	13 (7.3)
**Household income in soles**		
	300 to 1000 soles	28 (15.8)
	1001 to 2000 soles	65 (36.7)
	2001 to 3000 soles	22 (12.4)
	3001 to 5000 soles	31 (17.5)
	5001 to more	31 (17.5)
**Religion**		
	Catholic	143 (80.8)
	Non catholic	17 (9.6)
	None	17 (9.6)
Family members in the household *		4 (3–5)
**Alcoholism**		17 (9.8)
**Smoking**		11 (6.3)
**Comorbidity**		
	No	147 (84.5)
	Hypertension	4 (2.3)
	Diabetes	2 (1.2)
	Obesity	14 (8.1)
	Other	7 (4.0)
**Personal mental health history**		12 (6.8)
**Family history of mental health**		20 (11.3)
**Location at the time of the earthquake**		
	House	134 (75.7)
	Neighbor’s/friend’s house	5 (2.8)
	Place of work	15 (8.5)
	Public place	23 (13.0)
**Nervous breakdown immediately after earthquake**		69 (39.0)
**Physical injury caused by the earthquake**		5 (2.8)
**Family member with physical injury caused by the earthquake**		3 (1.7)
**Housing damage due to the earthquake**		
	Not affected	140 (79.1)
	Minor	34 (19.2)
	Moderate	2 (1.1)
	Severe	1 (0.6)
**Loss of job due to the earthquake**		4 (2.3)
**Social and/or material support**		
	Family/relatives	105 (59.3)
	Neighbors	38 (21.6)
	Friends	93 (52.8)
	Religious	17 (9.7)
	Politicians	11 (6.3)
	Government	10 (5.7)
	NGO	6 (3.4)
**Food insecurity**		
	Security	122 (68.9)
	Mild	29 (16.4)
	Moderate	10 (5.7)
	Severe	16 (9.0)
**Insomnia**		
	Absence	94 (55.6)
	Subclinical	59 (34.9)
	Moderate clinical	12 (7.1)
	Severe clinical	4 (2.4)
**Resilience**		
	Low	102 (59.0)
	High	71 (41.0)
**Anxiety**		
	No	85 (48.0)
	Mild	57 (32.2)
	Moderate	27 (15.3)
	Severe	8 (4.5)
**Depression**		
	Minimal	85 (48.3)
	Mild	50 (28.4)
	Moderate	25 (14.2)
	Moderate-severe	9 (5.1)
	Severe	7 (4.0)

* Median (25th percentile–75th percentile).

**Table 2:** Characteristics associated with depression and anxiety after the 6.1 magnitude earthquake in Piura.

Variables		*Depressive Symptoms*		p *	*Anxiety Symptoms*		*p* *
		No (*n* = 85)	Yes (*n* = 91)		No (*n* = 85)	Yes (*n* = 92)	
		N (%)	N (%)		N (%)	N (%)	
Age †**		23 (21–34)	21 (20–25)	0.006	22 (21–30)	21 (20–27)	0.042
**Sex**				0.140			0.124
	Female	42 (43.3)	55 (56.7)		42 (42.9)	56 (57.1)	
	Male	42 (54.5)	35 (45.5)		42 (54.6)	35 (45.5)	
**Single**		61 (43.9)	78 (56.1)	0.023	62 (44.3)	78 (55.7)	0.053
**Level of education**				0.511			0.344
	Secondary	22 (47.8)	24 (52.2)		18 (39.1)	28 (60.9)	
	Non-university higher education	11 (61.1)	7 (38.9)		10 (55.6)	8 (44.4)	
	University higher education	52 (46.4)	60 (53.6)		57 (50.4)	56 (49.6)	
**Current job**				0.136			0.043
	Worker	25 (50.0)	25 (50.0)		26 (52.0)	24 (48.0)	
	Household worker	2 (22.2)	7 (77.8)		1 (11.1)	8 (88.9)	
	Student	45 (46.4)	52 (53.6)		45 (45.9)	53 (54.1)	
	Unemployed	3 (42.9)	4 (57.1)		3 (42.9)	4 (57.1)	
	Others	10 (76.9)	3 (23.1)		10 (76.9)	3 (23.1)	
**Household income in soles**				0.075			0.206
	300 to 1000 soles	9 (32.1)	19 (67.9)		10 (35.7)	18 (64.3)	
	1001 to 2000 soles	30 (46.2)	35 (53.9)		31 (47.7)	34 (52.3)	
	2001 to 3000 soles	14 (66.7)	7 (33.3)		15 (68.2)	7 (31.8)	
	3001 to 5000 soles	19 (61.3)	12 (38.7)		16 (51.6)	15 (48.4)	
	5001 to more	13 (41.9)	18 (58.1)		13 (41.9)	18 (58.1)	
**Religion**				0.508			0.992
	Catholic	70 (49.3)	72 (50.7)		69 (48.3)	74 (51.8)	
	Non catholic	9 (52.9)	8 (47.1)		8 (47.1)	9 (52.9)	
	None	6 (35.3)	11 (64.7)		8 (47.1)	9 (52.9)	
Family members in the household ††***		4.49 ± 1.59	4.74 ± 2.10	0.392	4.48 ± 1.69	4.77 ± 2.02	0.284
**Alcoholism**		8 (47.1)	9 (52.9)	0.897	8 (47.1)	9 (52.9)	0.916
**Smoking**		5 (45.5)	6 (54.5)	0.832	4 (36.4)	7 (63.6)	0.414
**Comorbidity**		11 (40.7)	16 (59.3)	0.377	11 (40.7)	16 (59.3)	0.394
**Personal mental health history**		1 (8.3)	11 (91.7)	0.004	3 (25.0)	9 (75.0)	0.098
**Family history of mental health**		7 (35.0)	13 (65.0)	0.206	7 (35.0)	13 (65.0)	0.216
**Location at the time of the earthquake**				0.560			0.368
	House	61 (45.9)	72 (54.1)		63 (47.0)	71 (53.0)	
	Neighbor’s/friend’s house	2 (40.0)	3 (60.0)		1 (20.0)	4 (80.0)	
	Place of work	8 (53.3)	7 (46.7)		7 (46.7)	8 (53.3)	
	Public place	14 (60.9)	9 (39.1)		14 (60.9)	9 (39.1)	
**Physical injury caused by the earthquake**		1 (20.0)	4 (80.0)	0.199	0 (0.0)	5 (100.0)	0.029
**Family member with physical injury caused by the earthquake**		0 (0.0)	3 (100.0)	0.091	0 (0.0)	3 (100.0)	0.093
**Housing damage due to the earthquake**				0.024			0.027
	Not affected	75 (54.0)	64 (46.0)		75 (53.6)	65 (46.4)	
	Minor	10 (29.4)	24 (70.6)		10 (29.4)	24 (70.6)	
	Moderate	0 (0.0)	2 (100.0)		0 (0.0)	2 (100.0)	
	Severe	0 (0.0)	1 (100.0)		0 (0.0)	1 (100.0)	
**Loss of job due to the earthquake**		0 (0.0)	4 (100.0)	0.051	0 (0.0)	4 (100.0)	0.052
**Social and/or material support**							
	Family/relatives	47 (44.8)	58 (55.2)	0.254	50 (47.6)	55 (52.4)	0.897
	Neighbors	17 (44.7)	21 (55.3)	0.593	16 (42.1)	22 (57.9)	9.388
	Friends	41 (44.1)	52 (55.9)	0.206	45 (48.4)	48 (51.6)	0.979
	Religious	10 (58.8)	7 (41.2)	0.373	9 (52.9)	8 (47.1)	0.687
	Politicians	3 (27.3)	8 (72.7)	0.144	5 (45.5)	6 (54.6)	0.846
	Government	3 (30.0)	7 (70.0)	0.226	5 (50.0)	5 (50.0)	0.912
	NGO	2 (33.3)	4 (66.7)	0.447	4 (66.7)	2 (33.3)	0.360
**Food insecurity**				0.019			0.012
	Security	67 (55.4)	54 (44.6)		68 (55.7)	54 (44.3)	
	Mild	12 (41.4)	17 (58.6)		11 (37.9)	18 (62.1)	
	Moderate	3 (30.0)	7 (70.0)		3 (30.0)	7 (70.0)	
	Severe	3 (18.8)	13 (81.3)		3 (18.8)	13 (81.3)	
**Insomnia**				<0.001			<0.001
	Absence	65 (69.2)	29 (30.9)		65 (69.2)	29 (30.9)	
	Subclinical	17 (28.8)	42 (71.2)		17 (28.8)	42 (71.2)	
	Moderate clinical	1 (8.3)	11 (91.7)		1 (8.3)	11 (91.7)	
	Severe clinical	0 (0.0)	4 (100.0)		0 (0.0)	4 (100.0)	
**Resilience**				<0.001			<0.001
	Low	36 (35.3)	66 (64.7)		37 (36.3)	65 (63.7)	
	High	49 (69.0)	22 (31.0)		47 (66.2)	24 (33.8)	

* *p*-value of categorical variables calculated with Chi-Square test. ** *p*-value of categorical-numerical variables calculated with the U-test (Mann–Whitney). † Median—interquartile range. *** Mean ± standard deviation. †† *p*-value of categorical-numeric variables calculated with the student *t*-test.

**Table 3:** Factors associated with depressive symptoms after the 6.1 earthquake in Piura, using simple and multiple regression analysis.

Characteristics		Depressive Symptoms					
		Simple Regression			Multiple Regression		
		PR	95% CI	p *	PR	95% CI	p *
**Age**		0.97	0.96–0.99	**<0.001**	0.95	0.92–0.98	**0.002**
**Sex**							
	Female	Ref.					
	Male	0.80	0.64–1.01	0.057			
**Single**		1.60	1.11–2.30	**0.012**	1.02	0.55–1.90	0.940
**Level of education**							
	Secondary	Ref.					
	Non-university higher education	0.75	0.37–1.48	0.403			
	University higher education	1.03	0.86–1.22	0.769			
**Current job**							
	Worker	Ref.			Ref.		
	Household worker	1.56	1.19–2.03	**0.001**	1.35	0.90–2.01	0.142
	Student	1.07	0.90–1.28	0.446	0.60	0.44–0.82	**0.002**
	Unemployed	1.14	0.81–1.62	0.453	0.85	0.63–1.14	0.272
	Others	0.46	0.16–1.29	0.142	0.50	0.28–0.92	**0.026**
**Household income in soles**							
	300 to 1000 soles	Ref.			Ref.		
	1001 to 2000 soles	0.79	0.54–1.16	0.235	0.91	0.55–1.51	0.718
	2001 to 3000 soles	0.49	0.15–1.60	0.237	0.77	0.26–2.23	0.625
	3001 to 5000 soles	0.57	0.42–0.78	**<0.001**	0.72	0.46–1.14	0.163
	5001 to more	0.86	0.64–1.15	0.303	1.00	0.76–1.31	0.980
**Religion**							
	Catholic	Ref.					
	Non catholic	0.93	0.66–1.30	0.666			
	None	1.28	0.99–1.64	0.057			
**Family members in the household**		1.03	0.99–1.08	0.116			
**Alcoholism**		1.03	0.59–1.80	0.911			
**Smoking**		1.06	0.63–1.81	0.817			
**Comorbidity**		1.19	0.73–1.92	0.489			
**Personal mental health history**		1.88	1.34–2.63	**<0.001**	1.88	1.33–2.67	**<0.001**
**Family history of mental health**		1.30	1.03–1.64	**0.028**	0.99	0.85–1.16	0.895
**Location at the time of the earthquake**							
	House	Ref.					
	Neighbor’s/friend’s house	1.11	0.75–1.63	0.600			
	Place of work	0.86	0.45–1.65	0.654			
	Public place	0.72	0.47–1.12	0.148			
**Physical injury caused by the earthquake**		1.57	0.98–2.51	0.058			
**Family member with physical injury caused by the earthquake**		1.97	1.80–2.15	**<0.001**	0.54	0.40–0.74	**<0.001**
**Housing damage due to the earthquake**							
	Not affected	Ref.			Ref.		
	Minor	1.53	1.16–2.02	**0.002**	1.14	0.86–1.50	0.357
	Moderate	2.17	1.94–2.44	**<0.001**	2.02	1.12–3.65	0.019
	Severe	2.17	1.94–2.44	**<0.001**			
**Loss of job due to the earthquake**		1.98	1.83–2.14	**<0.001**	0.66	0.32–1.37	0.264
**Social and/or material support**							
	Family/relatives	1.19	0.85–1.66	0.313			
	Neighbors	1.10	0.92–1.30	0.294			
	Friends	1.21	0.95–1.53	0.118			
	Religious	0.78	0.42–1.48	0.453			
	Politicians	1.45	1.27–1.66	**<0.001**	2.33	1.22–4.45	**0.010**
	Government	1.39	1.12–1.73	**0.003**	0.63	0.36–1.10	0.105
	NGO	1.31	0.77–2.24	0.325			
**Food insecurity**							
	Security	Ref.			Ref.		
	Mild	1.31	0.94–1.84	0.114	1.40	0.95–2.06	0.090
	Moderate	1.57	0.85–2.90	0.152	1.73	1.07–2.80	**0.026**
	Severe	1.82	1.33–2.50	**<0.001**	1.49	0.83–2.67	0.182
**Insomnia**							
	Absence	Ref.			Ref.		
	Subclinical	2.31	1.58–3.37	**<0.001**	1.82	1.28–2.58	**0.001**
	Moderate clinical	2.97	2.34–3.77	**<0.001**	2.72	1.61–4.59	**<0.001**
	Severe clinical	3.24	2.62–4.02	**<0.001**	2.74	1.83–4.11	**<0.001**
**Resilience**							
	Low	Ref.			Ref.		
	High	0.48	0.31–0.74	**0.001**	0.53	0.42–0.68	**<0.001**

* *p*-values obtained with Generalized Linear Models (GLM), Poisson family, log link function, robust variance, and cluster by district.

**Table 4:** Factors associated with anxiety symptoms after the 6.1 earthquake in Piura, using simple and multiple regression analysis.

Characteristics		Anxiety Symptoms					
		Simple Regression			Multiple Regression		
		PR	95% CI	p *	PR	95% CI	p *
**Age**		0.98	0.96–1.00	0.069			
**Sex**							
	Female	Ref.					
	Male	0.80	0.59–1.07	0.126			
**Single**		1.47	0.72–3.02	0.290			
**Level of education**							
	Secondary	Ref.			Ref.		
	Non-university higher education	0.73	0.45–1.18	0.196	0.97	0.78–1.22	0.822
	University higher education	0.81	0.69–0.97	**0.019**	0.97	0.82–1.14	0.688
**Current job**							
	Worker	Ref.			Ref.		
	Household worker	1.85	1.35–2.53	**<0.001**	1.14	0.79–1.66	0.487
	Student	1.13	0.75–1.70	0.568	0.94	0.71–1.25	0.665
	Unemployed	1.19	0.75–1.90	0.465	0.87	0.42–1.78	0.697
	Others	0.48	0.14–1.65	0.244	0.56	0.27–1.18	0.127
**Household income in soles**							
	300 to 1000 soles	Ref.					
	1001 to 2000 soles	0.81	0.61–1.08	0.154			
	2001 to 3000 soles	0.49	0.17–1.44	0.198			
	3001 to 5000 soles	0.75	0.55–1.04	0.083			
	5001 to more	0.90	0.79–1.03	0.138			
**Religion**							
	Catholic	Ref.					
	Non catholic	1.02	0.67–1.56	0.916			
	None	1.02	0.62–1.70	0.930			
**Family members in the household**		1.04	0.97–1.12	0.275			
**Alcoholism**		1.03	0.55–1.90	0.934			
**Smoking**		1.25	0.76–2.06	0.383			
**Comorbidity**		1.18	0.83–1.67	0.361			
**Personal mental health history**		1.49	1.10–2.02	**0.009**	1.38	0.91–2.11	0.130
**Family history of mental health**		1.29	1.06–1.57	**0.010**	1.04	0.79–1.36	0.787
**Location at the time of the earthquake**							
	House	Ref.			Ref.		
	Neighbor’s/friend’s house	1.51	1.31–1.73	**<0.001**	1.74	0.95–3.17	0.072
	Place of work	1.01	0.61–1.65	0.979	0.65	0.34–1.24	0.191
	Public place	0.74	0.45–1.22	0.233	0.76	0.43–1.34	0.342
**Physical injury caused by the earthquake**		1.98	1.77–2.21	**<0.001**	2.69	1.85–3.93	**<0.001**
**Family member with physical injury caused by the earthquake**		1.96	1.72–2.22	**<0.001**	0.9	0.74–1.10	0.302
**Housing damage due to the earthquake**							
	Not affected	Ref.			Ref.		
	Minor	1.52	1.19–1.94	**0.001**	1.1	0.78–1.54	0.593
	Moderate	2.15	1.92–2.42	**<0.001**	0.58	0.22–1.51	0.267
	Severe	2.15	1.92–2.42	**<0.001**			
**Loss of job due to the earthquake**		1.97	1.76–2.20	**<0.001**	0.94	0.64–1.39	0.767
**Social and/or material support**							
	Family/relatives	1.02	0.89–1.17	0.780			
	Neighbors	1.16	0.94–1.42	0.161			
	Friends	1.00	0.84–1.19	0.966			
	Religious	0.90	0.57–1.44	0.663			
	Politicians	1.06	0.75–1.50	0.749			
	Government	0.97	0.56–1.66	0.898			
	NGO	0.64	0.36–1.12	0.114			
**Food insecurity**							
	Security	Ref.			Ref.		
	Mild	1.40	1.17–1.68	**<0.001**	1.49	1.09–2.03	**0.012**
	Moderate	1.58	0.82–3.05	**0.171**	1.83	0.89–3.77	0.103
	Severe	1.84	1.32–2.54	**<0.001**	1.44	0.96–2.16	0.078
**Insomnia**							
	Absence	Ref.			Ref.		
	Subclinical	2.31	1.65–3.22	**<0.001**	1.98	1.38–2.85	**<0.001**
	Moderate clinical	2.97	2.52–3.50	**<0.001**	2.97	2.17–4.05	**<0.001**
	Severe clinical	3.24	2.75–3.81	**<0.001**	3.29	2.55–4.24	**<0.001**
**Resilience**							
	Low	Ref.			Ref.		
	High	0.53	0.36–0.77	**0.001**	0.62	0.52–0.75	**<0.001**

* *p*-values obtained with Generalized Linear Models (GLM), Poisson family, log-link function, robust variance, and cluster by district.

## Data Availability

The dataset generated and analyzed during the current study is not publicly available because the ethics committee has not provided permission/authorization to publicly share the data but are available from the corresponding author on reasonable request.
